# Disease-relevant signalling-pathways in head and neck cancer: Taspase1’s proteolytic activity fine-tunes TFIIA function

**DOI:** 10.1038/s41598-017-14814-x

**Published:** 2017-11-02

**Authors:** Alena Gribko, Angelina Hahlbrock, Sebastian Strieth, Sven Becker, Jan Hagemann, Max Deichelbohrer, Andreas Hildebrandt, Negusse Habtemichael, D. Wünsch

**Affiliations:** 1 Department of Otorhinolaryngology, Molecular and Cellular Oncology, University Hospital of Mainz, Langenbeckstrasse 1, Mainz, 55101 Germany; 2 Scientific Computing and Bioinformatics, Johannes Gutenberg University, Staudingerweg 9, Mainz, 55128 Germany

## Abstract

Head and neck cancer (HNC) is the seventh most common malignancy in the world and its prevailing form, the head and neck squamous cell carcinoma (HNSCC), is characterized as aggressive and invasive cancer type. The transcription factor II A (TFIIA), initially described as general regulator of RNA polymerase II-dependent transcription, is part of complex transcriptional networks also controlling mammalian head morphogenesis. Posttranslational cleavage of the TFIIA precursor by the oncologically relevant protease Taspase1 is crucial in this process. In contrast, the relevance of Taspase1-mediated TFIIA cleavage during oncogenesis of HNSCC is not characterized yet. Here, we performed genome-wide expression profiling of HNSCC which revealed significant downregulation of the TFIIA downstream target CDKN2A. To identify potential regulatory mechanisms of TFIIA on cellular level, we characterized nuclear-cytoplasmic transport and Taspase1-mediated cleavage of TFIIA variants. Unexpectedly, we identified an evolutionary conserved nuclear export signal (NES) counteracting nuclear localization and thus, transcriptional activity of TFIIA. Notably, proteolytic processing of TFIIA by Taspase1 was found to mask the NES, thereby promoting nuclear localization and transcriptional activation of TFIIA target genes, such as CDKN2A. Collectively, we here describe a hitherto unknown mechanism how cellular localization and Taspase1 cleavage fine-tunes transcriptional activity of TFIIA in HNSCC.

## Introduction

Head and neck cancers (HNC) are among the most common malignant neoplasms in humans^1^. HNC is typically diagnosed at advanced stages with metastases resulting in a 5-year survival rate of less than 50%^2^. The prevailing form of HNC, head and neck squamous cell carcinoma (HNSCC), is characterized as a very aggressive and invasive cancer type affecting multiple sites of the upper aerodigestive tract like the nasal cavity, mouth, salivary glands, larynx and pharynx^2,3^. Major risk factors associated with the development of HNSCC are tobacco use, alcohol consumption and high-risk human papilloma virus infections (HPV)^4^. Due to the late disease presentation of the patient, lack of suitable biomarkers, and corresponding drugs for individually targeted therapy approaches, survival rates for HNSCC have not improved significantly within the last years^5–7^. Currently, the main prognostic parameters of HNSCC are the size and location of the tumour, the presence of distant metastasis and cervical lymph node metastases, which is not sufficient to evaluate the disease outcome^3,8,9^. Despite advances in therapy, the treatment of HNSCC still often comes along with functional impairment and cosmetic deformity of vital functions of the aerodigestive tract, such as breathing, swallowing, speech, hearing and smell^3^. Although the use of kinase inhibitors or antibodies has gained increasing clinical relevance, there is still urgent need for effective therapies and novel drug targets.

The protease Threonine Aspartase1 or Taspase1 has been identified as a promising new anti-cancer target which is critically involved in the development of aggressive infant leukaemias and HER2-associated breast cancer via its substrate MLL^10,11^. In addition, Taspase1 is overexpressed in a variety of solid tumours, including HNSCC^12^. The human *Taspase1* gene encodes a protein of 420 amino acids (aa) resembling the Taspase1 proenzyme. Based on structural similarities Taspase1 was classified as a type 2 asparaginase exhibiting several specific characteristics^13^. In contrast to the exclusively *cis*-active type 2 asparaginases, only Taspase1 is able to cleave distinct substrates in *trans* by hydrolyzing its target proteins at conserved (Q^3^[FILV]^2^D^1^↓G^1′^x^2′^D^3′^D^4′^) sites^14,15^. During mammalian development, Taspase1 plays an important role in the regulation of correct segmental identities, head morphogenesis and spermatogenesis^16–19^. However, the molecular mechanisms how Taspase1 may affect substrate functions through site-specific proteolysis still remain to be determined. Importantly, no specific small molecule or genetic inhibitors are available worldwide, hampering not only to further dissect Taspase1’s disease mechanisms, but also precluding the full assessment of its clinical impact^20–22^.

Besides MLL other essential proteins, such as the precursor of the transcription factor IIA (TFIIA) have been identified as native Taspase1 targets^23^. TFIIA has been initially characterized as part of the preinitiation complex initiating RNA polymerase II transcription^24^. TFIIA is composed of three subunits, α, β, and γ encoded by two separated genes, TFIIAαβ and TFIIAγ. The γ-subunit is conserved among different species, whereas sequence similarity in TFIIAαβ is limited mostly to the N-terminal region of the α-subunit and the C terminus covering most of the β-subunit^25^. TFIIAαβ is posttranslationally processed by Taspase1 at an evolutionary conserved cleavage site QVDG (aa 272 to 275)^23^. Interestingly, both uncleaved αβ and the cleaved α- and β-subunits can be found in association with the TFIIAγ subunit *in vivo* ^26,27^. Additionally, both complexes interact with the TATA-binding protein (TBP) on DNA and support transcription to similar extents *in vitro* and in reporter assays^28,29^. Therefore, uncleaved and cleaved forms of TFIIA may have distinct gene regulatory functions in differentiation. The observation that cleavage is the prerequisite for proteasome mediated degradation of TFIIA^28^ indicates that cleavage regulates TFIIA stability and thus, transcriptional activity. This hypothesis was supported by a study showing that Taspase1-mediated cleavage of TFIIA ensures proper head formation during mouse development^18^. It has been suggested that TFIIAαβ cleavage by Taspase1 results in suppression of CDKN2A expression and finally, in proper head formation^18^. The CDKN2A gene locus encodes for the cell cycle regulators p16^INK4a^, p19^ARF^ and p21^CIP^, blocking cell cycle progress in G1 and S phase^30^. Especially the tumour suppressor p16^INK4a^ is in focus of interest as putative biomarker for HNSCC.

Controlled distribution of macromolecules within different cellular compartments is an elaborated way of controlling protein activity. In eukaryotic cells, spatial and functional division is ensured by the nuclear envelope separating the nucleus from the cytoplasm^31^. Nucleocytoplasmic transport takes place through nuclear pores and is tightly regulated by specific signals and transport receptors^31^. In general, active nuclear import is mediated by short stretches of basic amino acids, termed nuclear localization signals (NLS), which interact with specific import receptors^12^. In contrast, signal mediated nuclear export pathways are less understood^32^. A well-characterized class of nuclear export signals (NES) consist of short leucine-rich stretches interacting with the export receptor Crm1^33^. Leucine-rich NES have been identified in an increasing number of cellular and viral proteins executing heterogeneous biological functions. Especially for transcription factors, executing their biological function within the cell nucleus, such regulated subcellular localization provides an attractive way to control their activity^34^. This indeed has been demonstrated for several key players of signal transduction cascades^35,36^.

Besides its relevance during normal head development, the molecular mechanisms how Taspase1 may affect TFIIA functions in malignant tissue of HNSCCs still remain to be determined. In order to close this knowledge gap, we combined gene expression analysis of HNSCC tumours with cell-based analysis of TFIIA localization and proteolytic cleavage. Although TFIIA was initially described as a nuclear transcriptional regulator^37^, we identified a significant fraction of TFIIA accumulating to the cytoplasm due to a highly efficient nuclear export signal. TFIIA´s ability to also reside in the cytoplasm not only counteracts its nuclear localization but also affects its transcriptional activity together with the proteolytic cleavage by Taspase1. Collectively, by regulating the nuclear availability of TFIIA, the interplay of nuclear export and Taspase1-mediated cleavage fine-tunes transcriptional regulation of TFIIA target genes, such as CDKN2A.

## Results

### Genome-wide expression profiling identifies CDKN2A downregulation in HNSCC tumours

**Figure 1:**
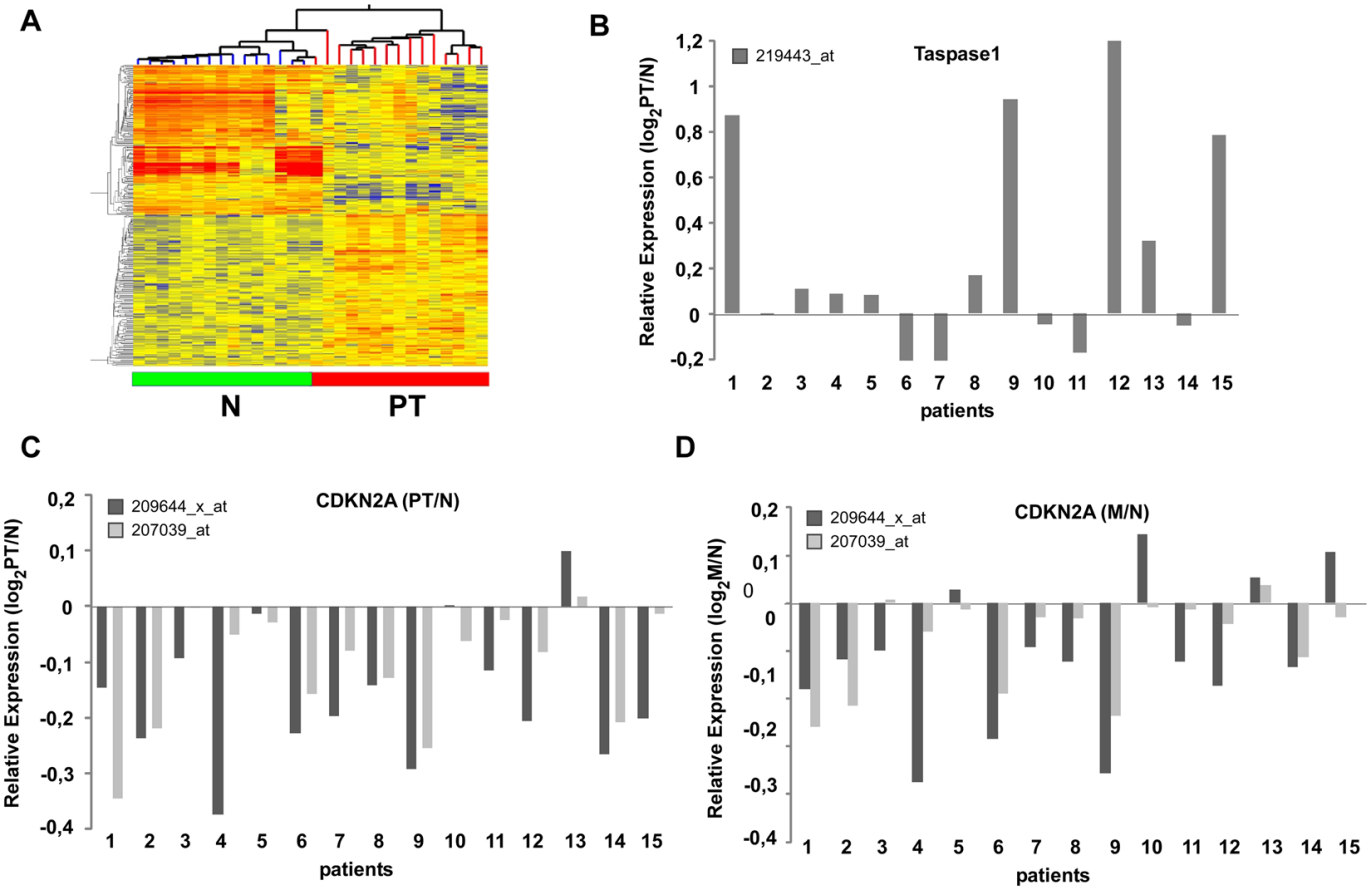
Genome-wide expression profiling of HNSCC primary tumours (PT) and lymph node metastases (M) versus normal tissue (N). (**A**) Intrinsic gene set cluster analysis of 45 HNSCC samples. Unsupervised two-way hierarchical clustering and gene tree representation of differentially regulated genes (fold change ≥ 2.0 and p-value < 0.05) allows to separate N and PT. X-axis represents patient samples; y-axis represents the list of probe sets grouped by similarity using Pearson correlation. The comparison of PT versus N revealed 650 deregulated genes. (**B**–**D**) Differential expression of Taspase1 (**B**) and CDKN2A (**C,D**) in 15 HNSCC tumours. Whereas the protease Taspase1 is overexpressed in primary tumours, CDKN2A expression is significantly downregulated in most PT (p-value < 0.0001) as well as M compared to N (p-value < 0.05). Relative expression levels (log_2_PT/N; log_2_M/N) obtained by *Affymetrix* microarray analysis are shown. CDKN2A locus is represented by two probe sets as indicated.

To identify genes differentially expressed in HNSCC primary tumours (PT) *versus* lymph node metastasis (M) and the corresponding non-malignant tissue (N), tissues were obtained from 20 patients undergoing surgical resection. In contrast to other studies, in which genetic and epigenetic variations of individual patients require the analysis of large patient cohorts to increase the relevance of the obtained data sets, our study design focused on a patient cohort from which N, PT as well as M could be surgically recovered from the same patient. All cases were diagnosed histopathologically and staged according to the TNM classification recommended by the UICC (for clinical parameters see Supplementary Table S1). RNA was isolated from snap-frozen biopsies and all samples were assayed using the *Affymetrix U133A 2.0* gene array. We excluded 5 patients from the study due to insufficient mRNA quality. Primarily a qualitative tool, we performed unsupervised average-linkage hierarchical clustering using the *GeneSpring* software and identified several groups/subtypes within the unfiltered cluster dendrogram (Fig. 1A, Supplementary Fig. S1). Non-malignant tissue and carcinomas grouped separately on a clustering dendrogram employing the complete datasets. Thus, HNSCC tumour tissue can be distinguished from corresponding non-malignant tissue and non-malignant tissue from lymph node metastases using even unsupervised RNA expression signatures. The comparison of PT versus N revealed 650 deregulated genes (Fig. 1A), whereas lymph node metastasis (M) versus N resulted in 1579 differentially expressed genes, and 393 genes in M versus PT (Supplementary Fig. S1).

As mentioned before, Taspase1-mediated cleavage of TFIIAαβ ensures proper head morphogenesis by suppressing expression of CDKN2A^18^. In order to investigate the relevance of Taspase1-TFIIA-CDKN2A signalling also in malignant tissue of the head, gene expression was compared in PT versus N as well as M versus N (Fig. 1). Interestingly, in 14 out of 15 patients CDKN2A was significantly downregulated in PT versus N represented by two probe sets (Fig. 1C; mean log_2_FC = 0.65, p value < 0.0001). Similar results were obtained for M versus N. Here, 11 out of 15 patients showed downregulation of CDKN2A (Fig. 1D; p value < 0.05). In contrast, expression of TFIIA was not significantly altered in the analysed cohort (data not shown). Another member of the signalling pathway, the protease Taspase1, was found to be overexpressed in the majority of the analysed patients (Fig. 1B), as demonstrated before^12^. However, the variation of Taspase1 gene expression was elevated in the present cohort including individual patients showing no regulation or even slight downregulation of Taspase1.

Upstream analyses of CDKN2A associated pathways using the Ingenuity Pathway Analysis tool support our hypothesis that in HNSCC patients CDKN2A pathways are inactivated which is attended by silencing of the negative cell cycle regulator RB1, the CDK inhibitor CDKN1A, and the tumour suppressor TP53 (Supplementary Fig. S2A). Of note, conflicting data involving TP53 as indicated by yellow lines in the network analysis may be explained by mutations of the TP53 gene frequently occurring in HNSCC tumours^38^. In concordance, upstream analysis of cell cycle-associated genes revealed that these genes are mainly activated (Supplementary Fig. S2B).

Conclusively, genome-wide expression analysis indicates that HNSCC carcinogenesis involves deregulated signalling pathways including Taspase1 and CDKN2A.

### TFIIA dynamically localizes in epithelial tumour cells

Although we found a significant downregulation of TFIIA-regulated CDKN2A locus in HNSCC patients, expression of TFIIA itself seems to be not altered. Besides transcriptional regulation, protein function could be also controlled by cellular localization and intracellular transport^39–41^. A prominent example is the group of nuclear factor-κB (NF-κB) proteins which regulate a variety of biological processes by translocating between cytoplasm and the nucleus^42^. Remarkably, TFIIA was initially described as a nuclear protein by the UniProtKB/Swiss-Prot database [P52655-TF2AA_HUMAN], but was also assigned to the cytoplasmic compartment by gene ontology [GO:0005737]. Here, we aim at the resolution of this inconsistency and the detailed characterization of TFIIA’s cellular localization in epithelial tumour cells.

**Figure 2:**
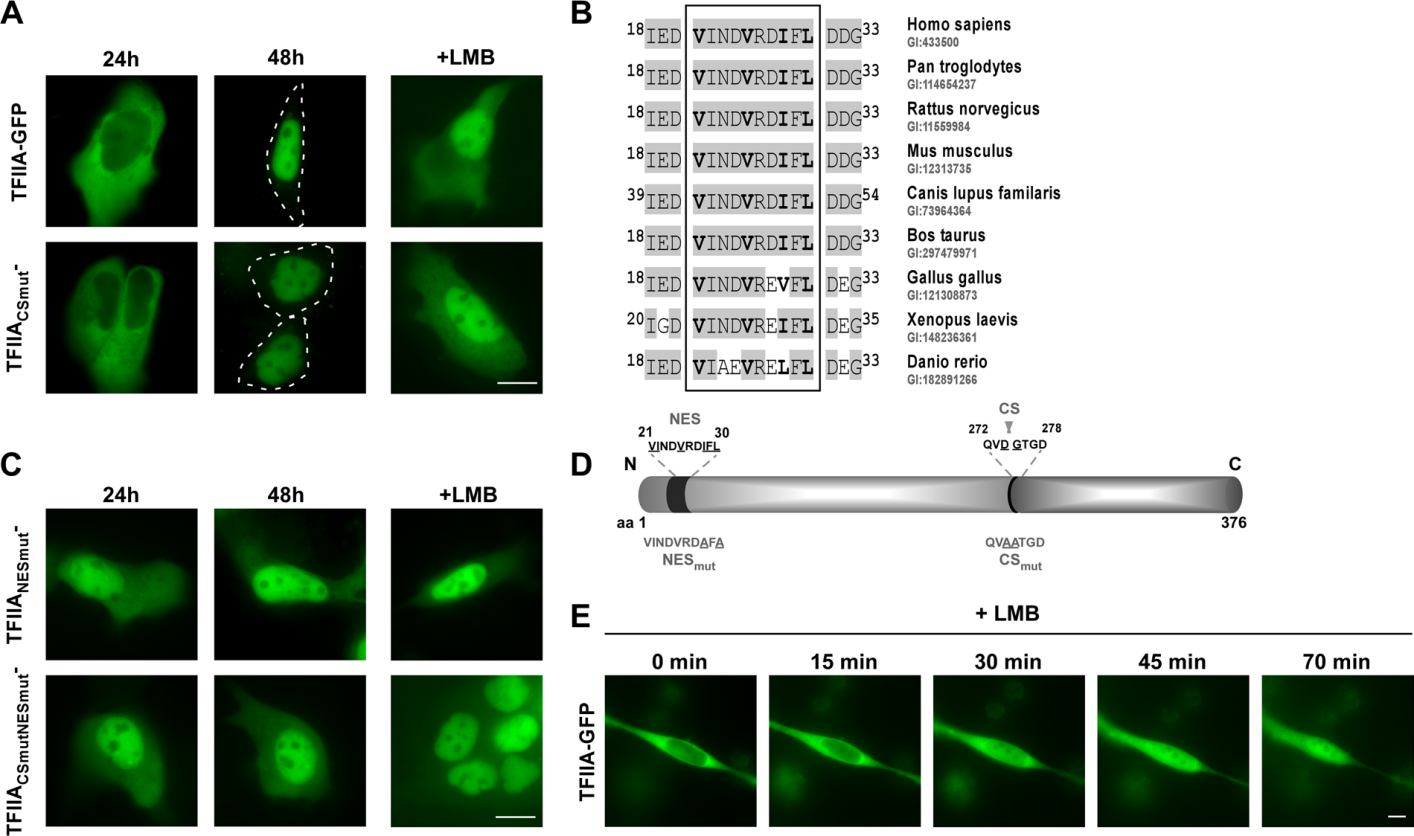
TFIIA dynamically localizes in epithelial tumour cells. (**A**) Transient transfection of A431 cells with wt TFIIA-GFP as well as the uncleaved mutant TFIIA_CSmut_-GFP revealed initial cytoplasmic localization after 24 h. TFIIA variants translocate to the nucleus within 48 h. Treatment with the nuclear export inhibitor LMB for 3 h leads to a rapid nuclear accumulation of TFIIA wt and TFIIA_CSmut_ variant. (**B**) TFIIA contains a putative nuclear export signal (aa ^21^ **VI**ND**V**RD**IFL** ^30^; hydrophobic aa in bold) which is evolutionary conserved among TFIIA orthologs. (**C**) In contrast to wt TFIIA, the TFIIANESmut- and CSmutNESmut-GFP fusion variants showed nuclear accumulation already 24 h after transfection which was neither significantly changed at later time points (48 h) nor by LMB treatment. (**D**) Schematic illustration of TFIIA domain organization indicating position and sequence of nuclear export signal (NES) and Taspase1 cleavage site (CS). For used mutants changes in the aa sequence are underlined. Size not drawn to scale. (**E**) TFIIA translocates from cytoplasm to nucleus after LMB treatment within 70 min. A431 cells were transfected with wt TFIIA-GFP and treated with the export inhibitor LMB 24 h after transfection. A single cell was captured every 5 minutes for at least 70 minutes. Scale bar, 10 µm.

To dissect the mechanisms regulating TFIIA’s intracellular localization, we cloned the *TFIIA*αβ open reading frame (ORF) from total RNA isolated from head and neck tumour tissue and confirmed its consistency with the ORF provided by official databases (UniProtKB - P52655). Since binding of the conserved TFIIAγ subunit does not differ dramatically between cleaved and uncleaved TFIIA forms, we focused on the analyses only of the TFIIAαβ precursor protein which will be termed TFIIA for convenience in the following text. In contrast to the reported nuclear localization, transient expression of TFIIA-GFP fusion protein revealed a predominant cytoplasmic localization in living interphase A431 cells after 24 h (Fig. 2A). Interestingly, TFIIA-GFP translocates from cytoplasm to nucleus within 48 h after transfection. As proteolytic cleavage by Taspase1 may play a critical role for TFIIA localization, we also analysed a cleavage-deficient TFIIA variant (TFIIA_CSmut_, ^272^QVDGTGD^278^ changed into ^272^QV**AA**TGD^278^, Fig. 2D). TFIIA_CSmut_-GFP showed similar localization characterized by initial cytoplasmic localization, which changed after 48 h to nuclear (Fig. 2A). As proteins larger than 40 kDa are unable to enter or exit the nucleus by passive diffusion but depend on active nuclear transport^43^, we analysed whether the dynamic translocation might be caused by active nuclear transport (Fig. 2B). Therefore, A431 cells were treated with the nuclear export inhibitor Leptomycin B (LMB) which specifically binds to the export receptor Crm1 and prevents its interaction with leucine-rich export signals^44^. Indeed, treatment with LMB lead to a rapid nuclear accumulation of TFIIA wt and TFIIA_CSmut_ suggesting the presence of an active, Crm1-dependent NES. Time-lapse experiments confirmed rapid nuclear accumulation of wt TFIIA within 70 minutes induced by LMB treatment (Fig. 2E). *In silico* analysis identified a hydrophobic region of the classical type (aa^21^ **VI**ND**V**RD**IFL** ^30^; hydrophobic aa in bold). Additionally, this motif is highly conserved in known TFIIA homologs (Fig. 2B), indicating a functional relevance of this putative NES.

To experimentally verify the activity of the NES, we cloned a TFIIA mutant (TFIIA_NESmut_) in which two essential hydrophobic amino acid residues in the putative export signal were changed into Ala (aa^21^ **VI**ND**V**RD**A** **F** **A** ^30^, changed aa underlined, Fig. 2D). Transient expression of TFIIA_NESmut-_GFP in A431 cells demonstrated that this mutation resulted in a nuclear accumulation of TFIIA directly after transfection, which was neither altered by prolonged transient expression nor by treatment with LMB (Fig. 2C). Combination of both mutations in the cleavage site and the NES (TFIIA_CSmutNESmut_) induces nuclear accumulation comparable to the single NES mutation (Fig. 2C). Importantly, dynamic localization of TFIIA variants could be also confirmed in the HNSCC cell line Fadu (Supplementary Fig. S3) and by automated cell analysis using the Cellomics ArrayScan Imaging Platform (Supplementary Fig. S4).

To finally prove that the predicted sequence indeed functions as a *bona fide* nuclear export signal, we performed microinjection experiments of recombinant transport substrates containing the described sequence (Supplementary Fig. S5). Whereas the N-terminal 40 aa residues of TFIIA mediated very fast and efficient export into the cytoplasm, mutation of the NES (aa^21^VINDVRDAFA ^30^) prevented nuclear export for at least 6 h. Collectively, these experiments unambiguously identified a leucine-rich NES in the α-subunit of TFIIA.

**Figure 3:**
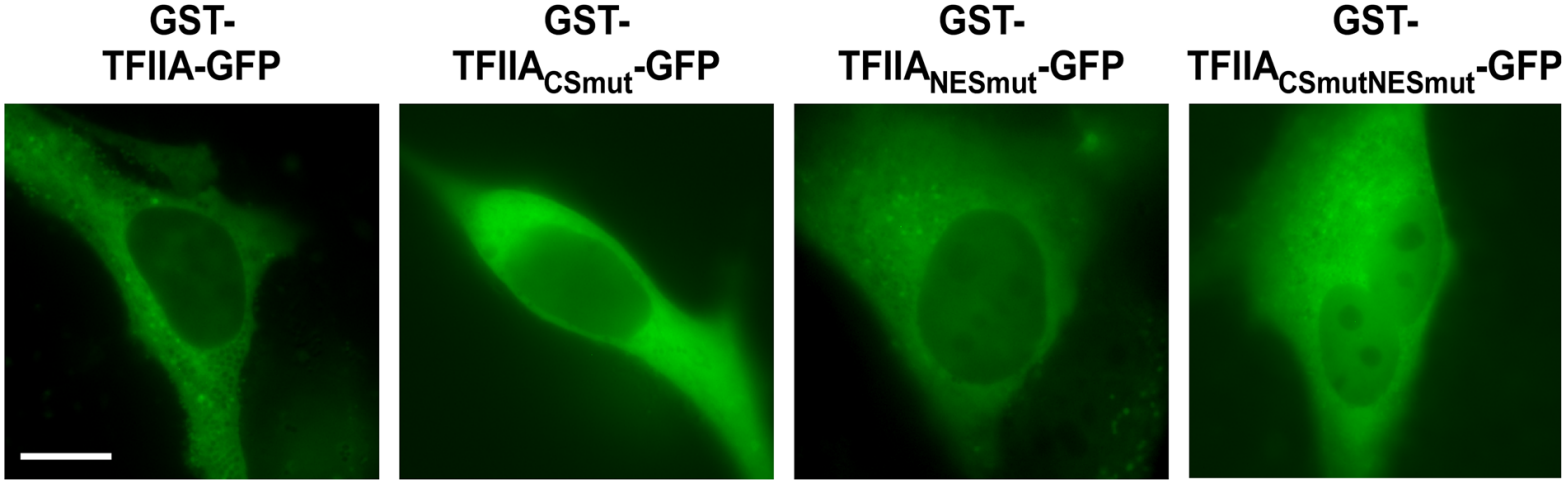
TFIIA is lacking an active nuclear import signal. Preventing passive diffusion of TFIIA-GFP variants by adding an N-terminal GST fusion tag results in an almost exclusively cytoplasmic localization. A431 cells were transfected with indicated constructs and visualized by fluorescence microscopy after 24 h. Scale bar, 10 µm.

Besides active nuclear export, nuclear accumulation of TFIIA might be enabled by an active nuclear localization signal (NLS) mediating importin-dependent nuclear import. In contrast to the results for the NES, bioinformatic analyses (NLStradamus, cNLS mapper, PredictProtein) employing regions of clustered basic amino acid residues as consensus sequence for NLS, did not indicate the presence of an active NLS in TFIIA (data not shown). In order to finally prove the absence of active nuclear import processes, we cloned diffusion-deficient TFIIA variants adding an N-terminal GST-tag to our constructs (Fig. 3). The resulting fusion proteins of approx. 88 kDa are not able to distribute within the cell by passive diffusion revealing active transport processes. Notably, for both NES-deficient mutants, TFIIA_NESmut_-GFP, TFIIA_CSmutNESmut_-GFP, a change of their prior nuclear localization to a cytoplasmic localization was detected, indicating deficiency in active nuclear access. In the absence of nuclear export, only active import signals could result in a nuclear accumulation of the diffusion-deficient GST fusion constructs. In summary, our results suggest that the dynamic cellular localization of TFIIA is actively regulated by its NES, but not by active nuclear import.

### TFIIA cleavage by Taspase1 regulates its subcellular localization and transcriptional activity

**Figure 4:**
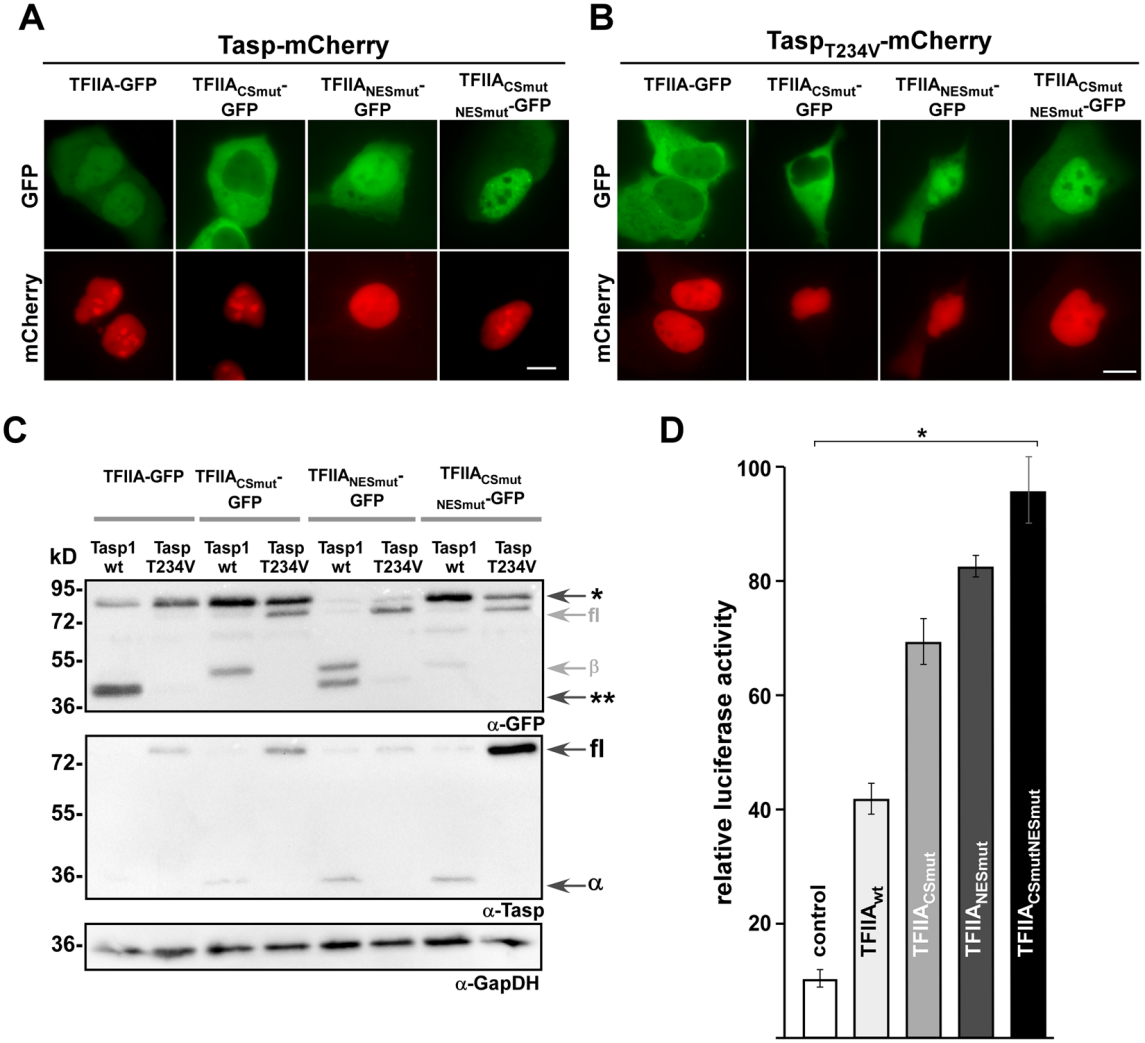
TFIIA cleavage by Taspase1 regulates its subcellular localization and transcriptional activity. (**A,B**) A431 cells were transfected with indicated TFIIA-GFP variants together with the active Taspase1- (**A**) or inactive Taspase_T243V_-mCherry (**B**) expression plasmid. Localization was analysed by fluorescence microscopy after 24 h. TFIIA-GFP is translocated from cytoplasm to nucleus after expression of active Taspase1 but not of inactive Taspase1_T234V_ mutant. In contrast the cleavage-deficient mutant TFIIA_CSmut-_GFP did not relocalize in presence of Taspase1. The export-deficient TFIIA variants did not alter their subcellular localization upon Taspase1 expression. Scale bars, 10 µm. (**C**) Proteolytic cleavage of TFIIA-GFP variants as shown by immunoblot analysis of whole-cell lysates. A431 cells were transfected with indicated TFIIA-GFP together the active Taspase1- (wt) or inactive Taspase1(T234V)- BFP expression plasmids. In contrast to TFIIA- and TFIIA_NESmut-_GFP, the TFIIA_CSmut_-GFP could not be cleaved by active wildtype (wt) Taspase1. The inactive Taspase1_T234V_ mutant neither showed self-cleavage in two active subunits nor *trans* cleavage activity of TFIIA. Expression of proteins and cleavage products in cell lysates was visualized using α-GFP and α-Taspase1 Abs. α-GapDH served as loading control. *Uncleaved TFIIA-GFP, **cleaved TFIIA-GFP, fl: Taspase1-BFP full length protein, α/β: Taspase1 α-/β-subunit. Of note, the α-GFP antibody is as well detecting the related BFP and thus full-length Tasp-BFP and the β-subunit containing the fusion tag. α-Taspase1 Ab is recognizing the full-length (75 kDa) as well as the α-subunit (28 kDa) of Taspase1. Shown blots are cropped. Full-length blots are shown in Supplementary Fig. S6. (**D**) Expression of the cell cycle regulator p16^INK4a^ is induced by impaired TFIIA export and proteolytic cleavage. A431 cells were co-transfected with either pGL3 basic or pGL3 basic-wt p16^INK4a^ 5′-UTR reporter, pRLSV40 and the respective TFIIA variant or pc3-GFP as negative control. Relative light units (RLU) were measured 48 h later, and plotted after normalization for transfection efficiency (pRLSV40). Bars, means of triplicates used to calculate standard deviations. Results of one representative experiment are shown, n = 3. Significance, *p < 0.005.

Although the cleavage-deficient TFIIA mutant showed localization similar to wt TFIIA, we wanted to undoubtedly characterize the dependence of TFIIA’s cellular localization on its proteolytic processing by Taspase1. Therefore, we co-expressed active Taspase1-mCherry (Fig. 4A) or its inactive mutant Tasp_T234V_-mCherry (Fig. 4B) with TFIIA-GFP variants. Transient co-expression of TFIIA-GFP with the fluorescently labelled active protease resulted in a nuclear accumulation of TFIIA already after 24 h (Fig. 3A). In contrast, the catalytically inactive mutant (Tasp_T234V_) did not alter TFIIA’s predominant cytoplasmic localization. As a control, co-expression of Taspase1 or its inactive mutant did not affect the cytoplasmic localization of the cleavage-deficient mutant TFIIA_CSmut_-GFP. Interestingly, the double mutant TFIIA_CSmutNESmut_ also did not change its localization neither by co-expression of active nor inactive Taspase1 (Fig. 4A,B). Cleavage of all TFIIA variants was also confirmed by immunoblot analysis (Fig. 4C). Of note, we observed that the expression of wt Tasp and the Tasp_T234V_ mutant could differ among samples. Since the expression plasmids are under the control of the same regulatory elements, these differences might be caused by altered protein stability of the uncleaved protease compared to cleaved subunits. However, the molecular details need to be confirmed in follow up studies.

It has been reported that Taspase1-mediated cleavage of TFIIA reduces expression of the CDK inhibitors p16^INK4a^ and p19^ARF^ encoded by the CDKN2A locus^18^. Since our results indicate that TFIIA cleavage by Taspase1 also diminishes active nuclear export of TFIIA, it is highly relevant if there is a link between TFIIA cleavage, subcellular localization and transcriptional activation of target genes, such as CDKN2A. To experimentally investigate such a putative link, we performed reporter gene studies using a p16^INK4a^-luciferase reporter construct (Fig. 4D). Therefore, A431 cells were co-transfected with TFIIA variants and reporter construct, and luciferase activity was measured with a dual luciferase assay using renilla luciferase for normalization. As expected, TFIIA wt efficiently induced transcription of the p16^INK4a^ reporter compared to co-transfection with an empty vector control. Furthermore, p16^INK4a^ expression was even more increased by the engineered TFIIA mutants exhibiting enhanced nuclear localization. Especially the combination of NES inactivation and Taspase1 cleavage-deficiency (TFIIA_CSmutNESmut_) resulted in a significant increase in reporter gene activation. Taken together, we could show that not only Taspase1-mediated cleavage of TFIIA, but also the integrity of its NES is able to regulate transcriptional activity of CDKN2A in epithelial cancer cells.

## Discussion

Focusing on disease-relevant signalling-pathways in head and neck cancer, the Taspase1-TFIIA-CDKN2A axis is an interesting and potentially druggable target for HNSCC. In the past it could be demonstrated that Taspase1 uses different strategies to regulate biological processes like proliferation, cell cycle, differentiation and apoptosis^11,19^. Interestingly, our data support the hypothesis that Taspase1 fine-tunes the transcriptional activity of TFIIA via its subcellular localization and proteolytic cleavage. Our study revealed that the TFIIAαβ precursor contains a biologically active nuclear export signal (NES, aa ^21^ **V**IND**V**RD**I**F**L** ^30^), but lacking a nuclear localization signal (NLS). The fact that transiently transfected, unprocessed TFIIA localized predominantly to the cytoplasm which was counteracted by treatment with the nuclear export inhibitor LMB, but unaffected by genetic inactivation of the Taspase1 cleavage site suggests accessibility of the NES in the uncleaved, full-length protein. On the other hand, nuclear import of TFIIA might be mediated via interaction with other proteins capable of nuclear import. For TFIIAγ, no NLS and thus no active nuclear import has been described so far. But there is evidence for other proteins to be shuttled inside different cellular compartments albeit lacking active transport signals. We discovered a comparable shuttling mechanism for the nuclear export of Taspase1 itself, making use of the NES of its interaction partner NPM1^12,45^.

With respect to proteolytic processing, overexpression of Taspase1 but not of catalytically inactive variants enhanced TFIIA’s direct nuclear accumulation, and uncleaved TFIIA mutants revealed an enhanced and elongated cytoplasmic localization. This leads us to the conclusion that processing of TFIIA by Taspase1 might mask the NES, albeit lacking support by the incomplete structural data available so far (pdb 1NVP). Possible scenarios include conformational changes in the α-subunit, which would shift the relative position of the subunit assembly and in turn lead to a complete masking of the NES. This could also be the result of interactions between TFIIA and other transcriptional factors, such as TBP.

Besides its crucial role for regulating the MLL-CyclinE axis in HER2/neu-positive breast cancer^10^, Taspase1 controls the expression of CDK inhibitors encoded by CDKN2A locus (p16^INK4a^ and p19^ARF^)^11,16,18,46^. Reporter gene studies revealed a transcriptional activation of p16^INK4a^ by TFIIA which was most prominent in TFIIA variants with genetically enforced nuclear localization in combination with impaired proteolytic cleavage. p16^INK4a^ is an important tumour suppressor gene, involved in the p16/cyclin-dependent kinase/retinoblastoma gene pathway of cell cycle control^47^. The gene encodes an inhibitor of CDK4 and CDK6, which regulate the phosphorylation of retinoblastoma gene and the G1 to S phase transition of the cell cycle^30^. p16^INK4a^ expression has been characterized in several tumour types ranging from its loss or downregulation to significant overexpression^48^. In head and neck cancer, p16^INK4a^ overexpression has been suggested to have a major impact on treatment response and survival in patients with HPV-positive tumours^49^. HPV-related cancers presenting p16^INK4a^ overexpression are very sensitive to radiotherapy, and have a better prognosis than HPV-negative tumours^50^. On the other hand, loss of CDKN2A expression by deletion, mutation, or hypermethylation is common in HNSCC and has been suggested as druggable target^51^. Although loss of p16^INK4a^ could not be validated as prognostic factor for HNSCC patients, it was suggested that it may be used to predict overall patient survival in early-stage head and neck tumours^30^. While genetic inactivation of p16^INK4a^ has been one of the most prominent genetic changes identified in human cancers to date, it has been reported that p16^INK4a^ function can be regulated via other complex cellular events, such as oncogene activation^52^. In our present study containing only HPV-negative HNSCC tumours, we found a moderate but highly significant downregulation of p16^INK4a^ suggesting rather expressional downregulation than complete loss or silencing of the CDKN2A gene. It has been reported that Taspase1-mediated cleavage of TFIIA affects the conformation of TFIID/TFIIA promoter complexes and thus, enables assembly with TFIID and other tissue-specific transcription factors^53^. Thereby, Taspase1-mediated cleavage of TFIIA regulates tissue-specific expression profiles, e.g. during spermatogenesis^16^ and mammalian head morphogenesis^18^, and is moreover essential for subsequent proteasomal degradation. The regulation of TFIIA protein levels via Taspase1-mediated cleavage is another regulatory key in this network allowing differential gene expression. In summary, this supports the hypothesis that in contrast to uncleaved TFIIAαβ/γ inducing bulk transcription, Taspase1 cleavage is regulating the switch between general and very specific transcriptional patterns and allows developmental fine-tuning. In undifferentiated cancer cells, Taspase1 activity might be regained and thus, originally developmental regulation processes might be reactivated. In summary, we propose a hitherto unknown mechanism how CDKN2A expression could be fine-tuned via the Taspase1-TFIIA signalling-pathway in head and neck tumours.

## Materials and Methods

### Antibodies (Ab), reagents and compounds

Ab used: α-TFIIA (sc-5314/sc-5315/sc-5311; Santa Cruz Biotechnology, Heidelberg, Germany); α-GAPDH (sc-47724; Santa Cruz Biotechnology, Heidelberg, Germany); α-GFP (sc-8334; Santa Cruz Biotechnology, Heidelberg, Germany); α-Taspase1 (sc-85945; Santa Cruz Biotechnology, Heidelberg, Germany). Appropriate HRP-, Cy3- or FITC-conjugated secondary antibodies (Sigma Aldrich, Munich, Germany; Santa Cruz Biotechnology, Heidelberg, Germany) were used. Reagents were from Sigma Aldrich (Sigma Aldrich, Munich, Germany) unless stated otherwise. Cells were treated with the export inhibitor Leptomycin B (LMB) (10 nM) as described^54^.

### Patient characteristics and material

Tissue samples were obtained from patients undergoing surgical resection at the departments of otolaryngology of the Universities of Frankfurt and Mainz. Investigation has been conducted in accordance with the ethical standards according to the Declaration of Helsinki and according to national and international guidelines. The study protocol has been approved by the ethics committees “Ethik-Kommission des Fachbereichs Medizin, Universitätsklinikum der Goethe-Universität” (#83756604) and “Ethik-Kommission der Landesärztekammer Rheinland-Pfalz” (#83748515) after obtaining the patients’ informed consent to participate in the study and was processed anonymously. All cases were diagnosed histopathologically as HNSCC and staged according to the TNM classification of malignant tumours recommended by the ‘Union International Contre le Cancer’ UICC. In this study, tumour specimens, corresponding non-malignant tissue, and lymph node metastasis were analysed. Specimens included oropharyngeal and laryngopharyngeal carcinoma of different tumour size (T1-T4), lymph node status (N0-2), no distant metastasis (M0) and grading G1-G3. Upon resection samples were immediately placed on ice and snap-frozen in liquid nitrogen within 30 min. Histological analyses were performed to ensure that each specimen contained >70% tumour tissue and <10% necrotic debris. Samples not meeting these criteria were rejected.

### RNA extraction

Frozen tissue samples (30–50 mg) were collected in 1ml Trizol (Invitrogen, Karlsruhe, Germany) and dispersed using an Ultra-Turrax T25 tissue homogenizer (IKA Werke, Staufen, Germany). Total RNA was extracted according to the recommendations given by the manufacturer’s Trizol protocol and further purified on RNeasy Mini spin columns (Qiagen, Hilden, Germany). Integrity and purity of total RNA were assessed on a Bioanalyzer 2100 (Agilent Technologies, Boeblingen, Germany) using a RNA 6000 Nano LabChip Kit (Agilent) according to the manufacturer’s instructions^55^.

### Target preparation and hybridization for Affymetrix GeneChip Arrays

Total RNA (5 µg) was used to prepare biotinylated cRNAs for hybridization, following the guidelines given in the Affymetrix GeneChip Expression Analysis Technical Manual. cRNA clean-up was performed on RNeasy Mini filters (Qiagen). In all, 10 µg of fragmented, labelled cRNA were hybridized to Affymetrix HG-U133A arrays (Affymetrix, Santa Clara, CA, USA) using standard conditions (16 h, 45 °C). Arrays were washed and stained in a Fluidics Station 400 (Affymetrix) and scanned on a Gene Array Scanner 2500 (Agilent), as recommended by Affymetrix. Raw fluorescence intensities from all hybridizations were normalized applying variance stabilization with additional scaling. Additionally, MAS5 as well as gcRMA expression values were calculated.

### Microarray data processing, pathway analysis and statistical analysis

Data and cluster analyses were performed using Affymetrix Microarray Suite 5.0 (MAS5) and GeneSpring GX software (Agilent Technologies, Santa Clara, USA). All samples were normalized to the median of control samples. Each measurement for each gene in those specific samples was divided by the median of that gene’s measurements in the corresponding control samples. The gene list was subjected to a student’s t-test (p-value < 0.05). The resulting list was further filtered for confidence using a Benjamini-Hochberg false discovery rate correction. A 2.0-fold cut-off filter was then applied to identify genes that were preferentially up- or downregulated. Heat-plots are used for visualizing gene expression: yellow indicates no change in expression, red enhanced expression (upregulated), and blue suppressed expression (downregulated).

The Ingenuity Pathway Analysis (IPA) tool (Ingenuity Systems) was used to identify pathways related to differentially expressed genes in primary tumours (PT*vs*.N). Data was filtered to meet the criteria p < 0.05 in our comparison analysis.

Analysis of the data was performed using R 2.15.2 with the *limma* package 3.14.4. Raw fluorescence intensities from all hybridizations were normalized, applying robust multichip average (RMA) normalization for the.CEL data, followed by a quantile normalization to compare expression results across specimens. For the comparison of primary tumours to normal mucosa (PT*vs*.N), and metastasis to normal mucosa (M*vs*.N) of differentially expressed genes, we also utilized the *limma* package. For multiple testing a Bonferroni correction was performed.

### Cell culture, microscopy fluorescence imaging of cells and computer-assisted microinjection

Cell lines used in the study were maintained and transfected as described^20,56^. Twelve-bit black and white images were captured using a digital Axiocam CCD camera (Carl Zeiss, Jena, Germany). Quantitation, image analysis and presentation were performed as described^33,57^. All assays were performed in triplicates. Purification and microinjection of recombinant GST-GFP transport substrates were performed as described in detail^33^. Automated analysis of the molecular translocation assay was performed using the Cellomics ArrayScan^®^ VTI Imaging Platform (Thermo Fisher Scientific Inc., Berkshire, UK) as described^58^. Scans were performed sequentially with settings to give sub-saturating fluorescence intensity, and a minimum of 400 valid objects per well was recorded.

### Plasmids

Expression constructs encoding TFIIA, Taspase1 and Taspase1_T234V_ as untagged or fusions with autofluorescent proteins were described^12^. Plasmids encoding TFIIA variants were amplified from full length TFIIA cDNA and cloned into pc3-GFP or pGex-GFP using BamHI/NheI-restriction sites. Plasmid TFIIA_CSmut_-, TFIIA_NESmut_-, and pGex_TFIIA_NESmut_-GFP were generated by splice overlap extension polymerase chain reaction as reported^59^. pF143 were described^60^. Plasmids were verified by sequence analysis^57^.

### Protein extraction, immunoblot analysis and immunofluorescence

Preparation of whole cell lysates was carried out as described using a physiological lysis buffer (50 mM Tris pH 8.0, 150 mM NaCl, 5 mM EDTA, 0.5% NP-40, 1 mM DTT, 1 mM PMSF, Complete Protease Inhibitory Cocktail from Roche Diagnostics, Mannheim, Germany). Equal loading of lysates was controlled by reprobing blots for GAPDH as described^61^. Immunofluorescence was performed as reported in detail^14,36^. Western blot data shown are representative for at least three independent experiments (n = 3).

### Reporter gene assays

For luciferase reporter gene assays, 5 × 10^4^ 293T cells seeded in 24-well plates 24 h before transfection. Cells were transfected at 80% confluence using PEI (2.7 µl PEI per µg DNA) as described^1^. Specifically, 350 ng of either the empty pGL3 basic or the pGL3 basic reporter vector containing the wild-type p16INK4a 5’-UTR were used along with 50 ng of the control pRLSV40 plasmid introduced to normalize transfection efficiency and 200 ng of the respective TFIIA variants or pc3-GFP as negative control. Cells were harvested 24 h after transfection, and luciferase assays were carried out using the dual-luciferase reagent (Promega, Madison, WI, USA). Briefly, relative light units (RLU) were measured for both luciferases, firefly and renilla, normalized for transfection efficiency and plotted as relative luciferase activity for each TFIIA variant. Assays were performed in triplicate, and data shown are representative for at least three independent experiments (n = 3).

### Bioinformatics

TFIIA proteins were analysed using NES-Finder (http://research.nki.nl/fornerodlab/NES-Finder.htm)^54^. Alignments and clustalW analyses were performed using BLAST (http://www.blast.ncbi.nlm.nih.gov/) and the BioEdit sequence alignment editor (http://www.mbio.ncsu.edu/BioEdit) with the human TFIIA amino acid sequence on NCBI databases (homo sapiens [GI: 433500]; bos taurus [GI: 297479971]; canis lupus familaris [GI: 73964364]; danio rerio [182891266]; gallus gallus [GI: 121308873]; mus musculus [GI: 12313735]; pan troglodytes [GI: 114654237]; rattus norvegicus [GI: 11559984]; xenopus laevis [GI: 148236361. Unless stated otherwise, data were obtained from three independent experiments done in triplicate.

### Data availability statement

The datasets generated during and/or analysed during the current study are available from the corresponding author on reasonable request.

## Electronic supplementary material

Supplementary Information
