# Supplementary material for: Disease-relevant signalling-pathways in head and neck cancer: Taspase1’s proteolytic activity fine-tunes TFIIA function
Source: Sci Rep. 2017 Nov 2;7:14937. doi: 10.1038/s41598-017-14814-x (PMC5668323; doi:10.1038/s41598-017-14814-x)
Supplement: Supplementary file 1 — Supplementary Information [file 41598_2017_14814_MOESM1_ESM.pdf]

Supplementary Information

Disease-relevant signalling-pathways in head and neck cancer: Taspase1´s proteolytic activity fine-tunes TFIIA function

Alena Gribko, Angelina Hahlbrock, Sebastian Strieth, Sven Becker, Jan Hagemann, Max Deichelbohrer, Andreas Hildebrandt, Negusse Habtemichael, and Désirée Wünsch

Supplementary Table S1: Overall clinical characteristics of HNSCC patients.

#	N/PT/M	Primary site	Age	Sex	HPV	pT	pN	Grading
**1**	N1/PT1/M1	Hypopharynx	47	F	-	2	2	2
**2**	N2/PT2/M2	Oropharynx	58	F	-	3	2	2
**3**	N3/PT3/M3	Hypopharynx	46	M	-	4	1	2
**4**	N4/PT4/M4	Larynx	68	M	-	4	2	2
**5**	N5/PT5/M5	Oropharynx	56	M	-	3	2	2
**6**	N6/PT6/M6	Hypopharynx	48	M	-	3	1	2
**7**	N7/PT7/M7	Larynx	56	M	-	4	3	3
**8**	N8/PT8/M8	Hypopharynx	58	M	-	3	2a	2
**9**	N9/PT9/M9	Hypopharynx	72	M	-	3	2	2
**10**	N10/PT10/M10	Oropharynx	49	M	-	3	1	2
**11**	N11/PT11/M11	Hypopharynx	57	M	-	1	2	3
**12**	N12/PT12/M12	Oropharynx	53	M	-	2	2b	2
**13**	N13/PT13/M13	Hypopharynx	57	M	-	2	3	2
**14**	N14/PT14/M14	Hypopharynx	47	M	-	2	2	2
**15**	N15/PT15/M15	Hypopharynx	56	M	-	2	3	2
16*	*PT16*	*Larynx*	*49*	*M*	-	*3*	*1*	*2*
17*	*N17/PT17*	*Hypopharynx*	*75*	*F*	-	*1*	*2*	*1*
18*	*PT18*	*Oropharynx*	*53*	*M*	-	*2*	*2*	*2*
19*	*PT19/M19*	*Hypopharynx*	*63*	*M*	-	*2*	*3*	*2*

*excluded from the study due to low RNA quality.

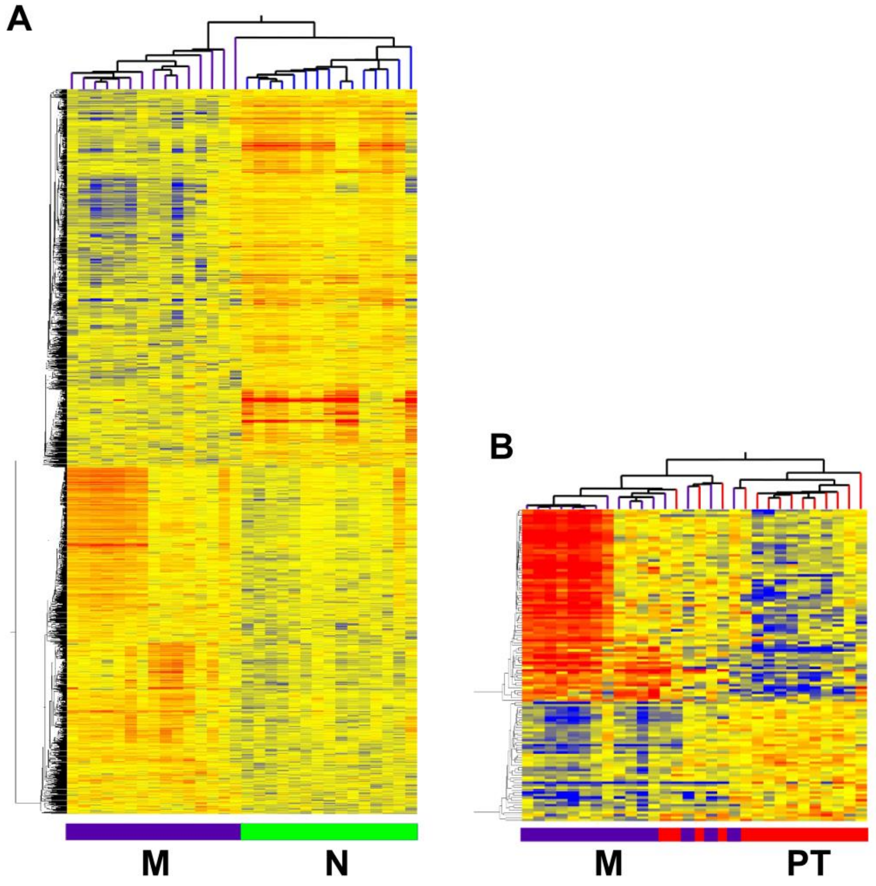

Supplementary Figure S1: Intrinsic gene set cluster analysis of 45 HNSCC samples allows profiling disease progression. Unsupervised two-way hierarchical clustering and gene tree representation of differentially regulated genes (fold change ≥ 2.0 and p-value < 0.05) allow to separate N, PT, and lymph node M. X-axis represents patient samples; y-axis represents the list of probe sets grouped by similarity using Pearson correlation. The comparison of lymph node metastasis (M) versus N resulted in 1579 differentially expressed genes (A). 393 genes were deregulated in M versus PT (B). Blue: downregulated. Red: upregulated. Yellow: unchanged.

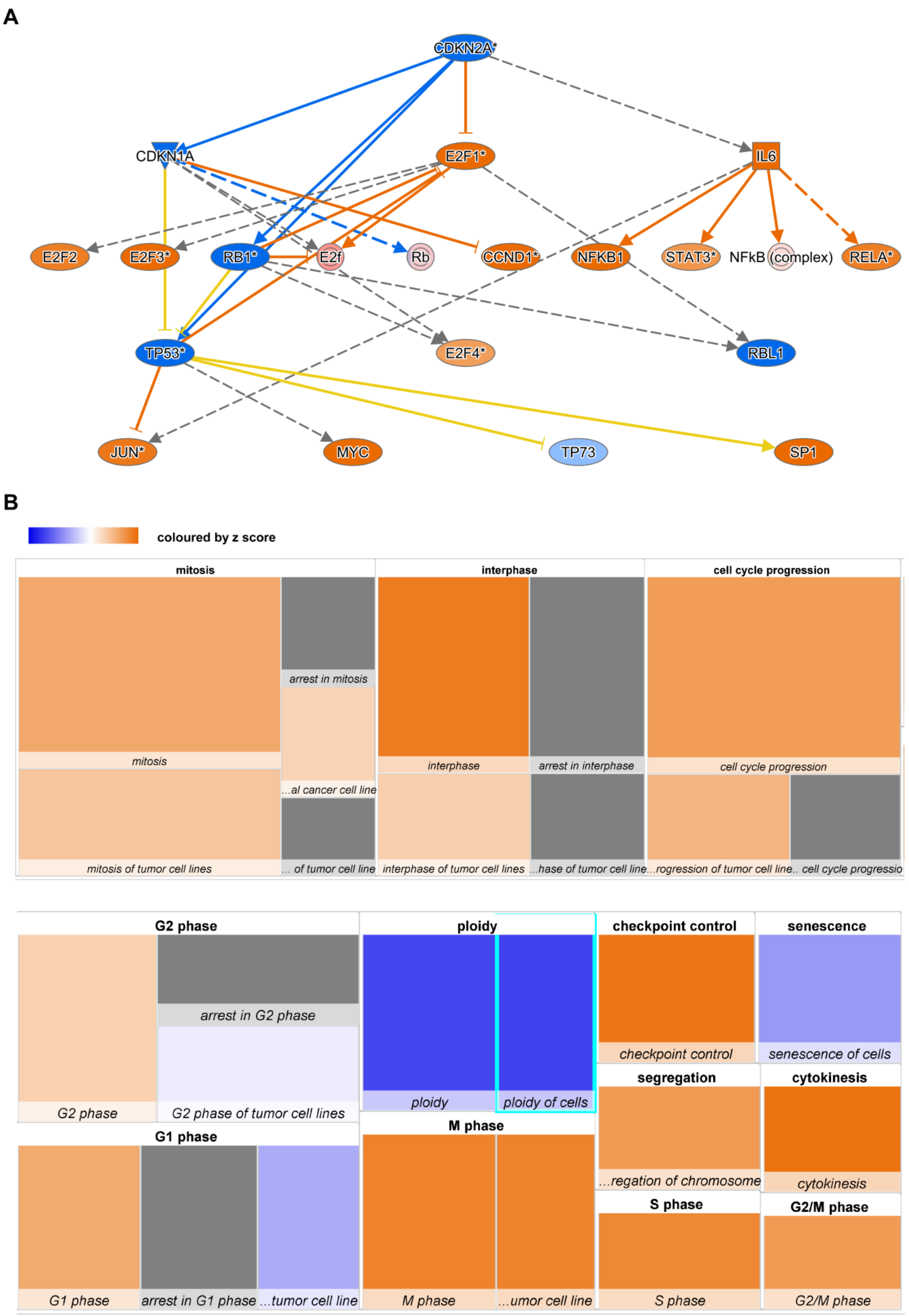

Supplementary Figure S2: Upstream analysis of CDKN2A associated network in PT*vs.*N (A) and cell cycle associated genes (B) by Ingenuity Pathway Analysis. A. For network analyses a p-value < 0.05 was used. Node colour indicates activated (orange), and inactivated (blue) genes. Lines and arrows between nodes represent direct (solid lines) and indirect (dashed lines) interactions between molecules. Yellow lines indicate conflicting data compared to expected regulation. All edges are supported by at least one reference from the literature or from canonical information stored in the Ingenuity Knowledge Base. Node shapes represent functional classes of gene products: triangle →

kinase, square → cytokine, concentric circle → group or complex, horizontal ellipse→ transcription regulator.

B. Heatmap produced by the upstream analysis tool of Ingenuity Pathway Analysis revealed activation of cell cycle pathways. All genes associated with cell cycle regulation were coloured by z-score, orange indicates activation, blue inactivation. Grey indicates no change.

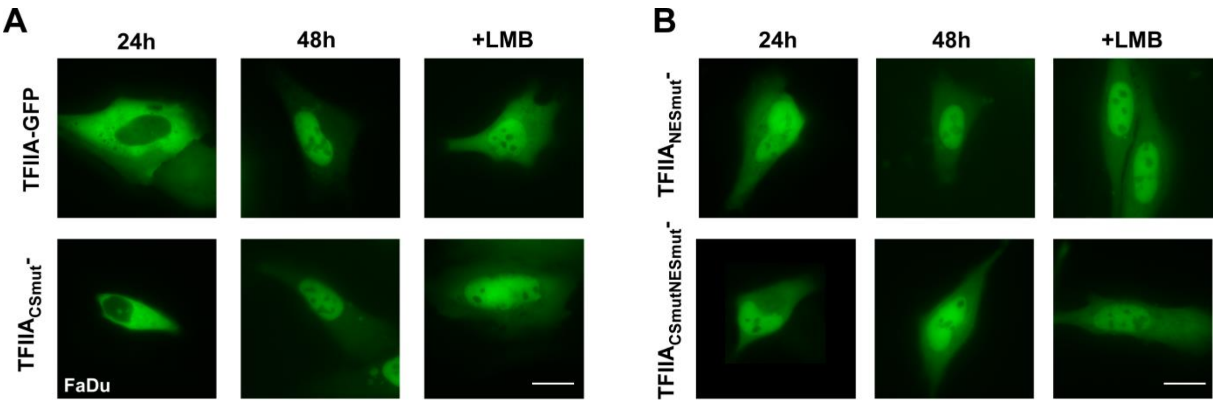

Supplementary Figure S3: TFIIA dynamically localizes in HNSCC tumour cells. A. Transient transfection of HNSCC Fadu cells with wt TFIIA-GFP as well as the uncleaved mutant TFIIA$_{CSmut}$-GFP revealed initial cytoplasmic localization after 24h. TFIIA variants translocate to the nucleus within 48 h. Treatment with the nuclear export inhibitor LMB for 3h leads to a rapid nuclear accumulation of TFIIA wt and TFIIA$_{CSmut}$ variant. B. In contrast to wt TFIIA, the TFIIA$_{NESmut}$- and CSmutNESmut-GFP fusion variants showed nuclear accumulation already 24h after transfection which was neither significantly changed at later time points (48h) nor by LMB treatment.

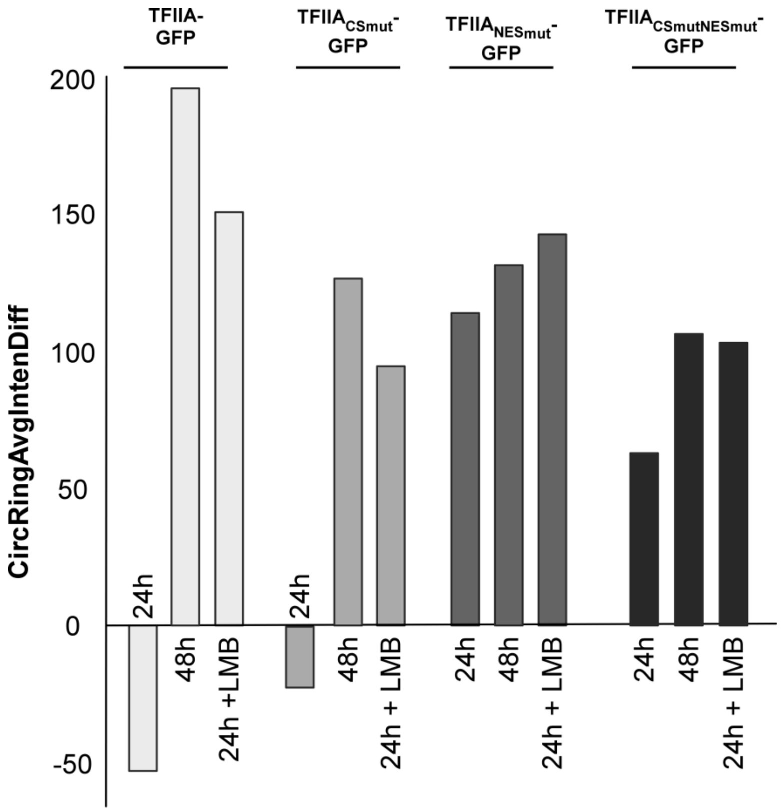

Supplementary Figure S4: Automated microscopic quantification of transfected A431 cells after 24h, 48h and after LMB treatment confirmed the dynamic TFIIA localization. After the analysis of at least 400 cells per well using the Cellomics ArrayScan, cytoplasmic and nuclear fluorescent intensity ratios were calculated from the circular mask representing the cell nucleus (CIRC), and the ambient cytoplasmic mask (RING). The calculated average intensity differences (CircRingAvgIntenDiff) are shown, where negative values equal a cytoplasmic, positive values a nuclear localization.

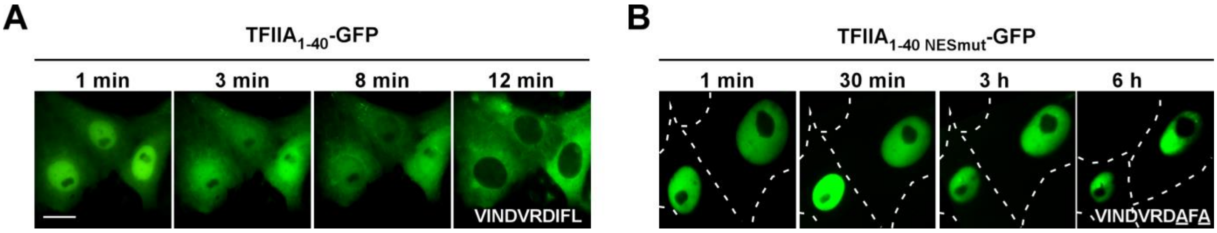

Supplementary Figure S5: The N-terminal domain of TFIIA contains an active nuclear export signal (NES). A./B. Indicated recombinant GST-TFIIA-GFP substrates were microinjected into the cytoplasm of Vero cells, and nuclear export documented in live cells by fluorescence microscopy after indicated time points. The N-terminal 40 aa residues of TFIIA mediated very fast and efficient export into the cytoplasm (A.), whereas mutation of two essential hydrophobic aa residues into alanine within the NES consensus (^{21}VINDVRD<u>A</u>F<u>A</u>^{30}; hydrophobic aa of the NES consensus in bold, mutated aa underlined) prevented nuclear export for at least 6 h (B.). Approximately 50 cells were injected, and representative examples are shown.

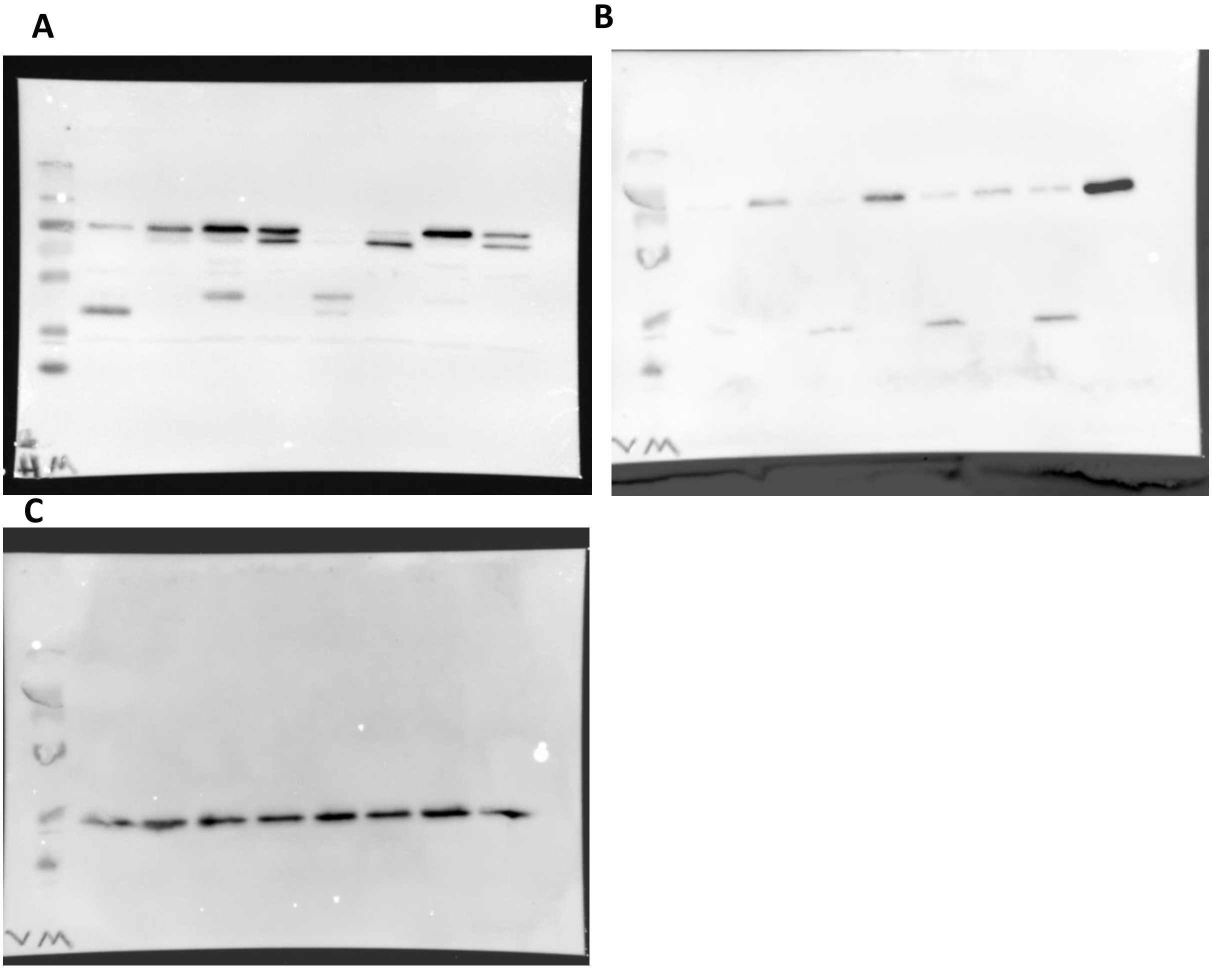

Supplementary Figure S6: Proteolytic cleavage of TFIIA-GFP variants as shown by immunoblot analysis of whole-cell lysates. Full-length blots referring to manuscript Figure 4C. Expression of proteins and cleavage products in cell lysates was visualized using α-GFP (A), α-Taspase1 (B), and α-GAPDH (C) Abs. Signals shown in C. are obtained from the same blot shown in B. after stripping and reprobing with α-GAPDH Ab.
